# *ACAT-1* gene polymorphism is associated with increased susceptibility to coronary artery disease in Chinese Han population: a case-control study

**DOI:** 10.18632/oncotarget.21649

**Published:** 2017-10-06

**Authors:** Yong-Tao Wang, Ying-Hong Wang, Yi-Tong Ma, Zhen-Yan Fu, Yi-Ning Yang, Xiang Ma, Xiao-Mei Li, Dilare Adi, Fen Liu, Bang-Dang Chen

**Affiliations:** ^1^ Department of Cardiology, First Affiliated Hospital of Xinjiang Medical University, Urumqi 830054, P.R. China; ^2^ Xinjiang Key Laboratory of Cardiovascular Disease Research, Urumqi 830054, P.R. China

**Keywords:** coronary artery disease, ACAT-1 gene, polymorphism, susceptibility, association studies

## Abstract

Several studies suggest an important role of Acyl-CoA: cholesterol acyltransferase-1(ACAT-1) in the development of atherosclerosis. The aim of present study was to investigate whether there exists a possible correlation between genetic variations in ACAT-1 genes and coronary artery disease (CAD) risk. Four polymorphisms (rs1044925, rs11545566, rs12121758 and rs10913733) were finally selected and genotyped in 750 CAD patients and 580 health controls, using the improved multiplex ligation detection reaction (iMLDR) method. We found that the rs11545566 G allele was associated with a significantly elevated CAD risk [GG vs. AA: adjusted odds ratio (AOR) = 1.62, 95% confidence interval (CI) = 1.13-2.32, P = 0.008; GA/GG vs. AA: AOR = 1.67, 95% CI = 1.22-2.29, P = 0.001]. The rs10913733 G allele was also associated with a significantly elevated CAD risk (GG vs. TT: AOR = 1.57, 95% CI = 1.08-2.28, P = 0.018; GT/GG vs. TT: AOR = 1.39, 95% CI = 1.07-1.79, P = 0.013). Multivariate linear regression analysis showed that the rs11545566 polymorphism was independently associated with the Gensini scores (P = 0.005). The Gensini score of subjects in the variant GG genotype group and the GG/GA genotype group were higher than the score of subjects in the AA genotype group (32.49 ± 26.60 and 31.26 ± 26.96 vs. 23.45 ± 21.64; P = 0.001 and 0.002, respectively). Our results demonstrate that ACAT-1 rs1154556 and rs10913733 polymorphism are novel genetic factors in the development of CAD. Rs11545566 was also associated with the severity of CAD.

## INTRODUCTION

Coronary artery disease (CAD) is the leading cause of death, responsible for about one in every five deaths worldwide [1–2]. Besides the conventional and modifable risk factors, a large number of studies have demonstrated that CAD is a complicated polygenic disease [3–7]. Studies on monozygotic twins estimated CAD heritability as high as 40%-60%. Up to now, more than 60 susceptibility locis for CAD have been identified [8].

Cholesterol is present in mammalian cell membranes and is essential for the growth and viability of cells. Excess cellular cholesterol is stored as cholesteryl esters (CEs). In most cases, CEs exists in the form of cytoplasmic lipid droplets and are present only in low levels. Chronic accumulation of CE in macrophages leads to the appearance of foamy and is a hallmark of early-stage atherosclerosis [9]. The cellular cholesterol homeostasis is highly regulated by various mechanisms. The enzyme ACAT, also known as acyl-coenzyme A (CoA): cholesterol acyltransferase plays an important role in cellular cholesterol homeostasis [10]. There are two isozymes of ACAT, ACAT-1 and ACAT-2, with different intracellular localization, membrane topology in mammalian species, and metabolic function for each enzyme [11–12]. To prevent over-accumulation at cellular membranes, ACAT converts free cholesterol to CEs and thereby controls the ratio of cellular free cholesterol and CEs [10–12]. ACAT-1 is widely expressed in various tissues and cells, and is responsible for the formation of foam cell in macrophages [13–15]. Previous studies also demonstrated that ACAT-1 was involved in the formation of atherosclerotic plaques, and thus might be a promising target for atherosclerosis and hypercholesterolemia treatment [16–17].

As far as we know, the genetic evidence on the association between ACAT-1 polymorphisms and CAD is poor. Ohta T et al found that -77G > A variant in ACAT-1 gene were significantly associated with plasma concentrations of HDL-C and apoA-I [18]. Dong-Feng Wu et al found that ACAT-1 rs1044925 may modulate serum HDL-C level and was associated with the risk of CAD [19]. All these suggested that ACAT-1 gene polymorphisms may play an important role in the formation of CAD. The primary aim of this analysis was to determine whether exists the relationship between polymorphisms of ACAT-1 gene and CAD in the Chinese Han population.

## RESULTS

### Participant characteristics

Demographic and clinical characteristics of all participants are summarized in Table 1. A total of 750 patients with CAD and 580 healthy controls were enrolled in this case-control study. Of the CAD patients, 211 (28.1%) were women and 539 (71.9%) were men, with mean age 61.22±10.55 years (range, 31-87 years). As to the controls, 179 (30.9%) were women and 401 (69.1%) were men, with mean age 60.66±11.01 years (range, 32-88 years). We did not observe significant differences between patients and controls regarding age (P = 0.341), sex (P = 0.278) and triglycerides (TG) (P = 0.071). The patients and controls differed significantly with regards to body mass index (BMI) (P = 0.048), total cholesterol (TC) (P < 0.001), high-density lipoprotein cholesterol (HDL-C) (P = 0.021), low-density lipoprotein cholesterol (LDL-C) (P < 0.001), glucose (P < 0.001), the prevalence of hypertension (P < 0.001), diabetes (P < 0.001), smoking (P < 0.001), and drinking (P < 0.001).

**Table 1:** Demographic and clinical characteristics of study participants

Characteristics	Control (n=580)	CAD (n=750)	χ2 or t	P value
Age, mean (SD)	60.66(11.01)	61.22 (10.55)	0.952	0.341
Sex, female (%)	179(30.9)	211(28.1)	1.175	0.278
Hypertension, n (%)	250(43.1)	480(64.0)	57.679	<0.001
Diabetes, n (%)	73(12.6)	236(31.5)	65.371	<0.001
Smoking, n (%)	106(18.3)	337(44.9)	104.629	<0.001
Drinking, n (%)	119 (20.5)	221(29.5)	13.766	<0.001
BMI, mean (SD)	25.07(3.29)	25.42 (3.09)	1.978	0.048
TG, mean (SD)	1.77 (1.07)	1.89(1.23)	1.805	0.071
TC, mean (SD)	4.16 (1.10)	4.77(1.16)	9.688	<0.001
HDL-C, mean (SD)	1.12 (0.34)	1.07(0.34)	2.309	0.021
LDL-C, mean (SD)	2.56 (0.83)	2.91 (0.94)	6.967	<0.001
Glucose, mean (SD)	5.49 (1.85)	6.21 (2.75)	5.417	<0.001

BMI, body mass index; TG, triglyceride; TC, total cholesterol; HDL-C, high density lipoprotein cholesterol; LDL-C, low density lipoprotein cholesterol; The *P* value of the continuous variables was calculated by the independen t-sample t-test. The *P* value of the categorical variables was calculated by χ2 test.

*p* < 0.05, statistical significance.

### Association between *ACAT-1* gene polymorphisms and risk and severity of CAD

The genotypes distribution of the four selected polymorphisms in patients with CAD and healthy controls are showed in Table 2. The genotype distributions of the four SNPs in controls were in accordance with the Hardy-Weinberg equilibrium (P = 0.934 for rs1044925, P = 0.169 for rs11545566, P = 0.373 for rs12121758 and P = 0.277 for rs10913733). The genotype frequencies of rs11545566 (17.0% for AA, 50.3% for GA and 32.7% for GG) among CAD patients were significantly different from those (22.9% for AA, 46.9% for GA and 30.2% for GG) among health controls (P = 0.028). After adjustment for age, sex, hypertension, diabetes, smoking, drinking status, BMI, TG, TC, HDL-C, LDL-C and Glucose, carriers of rs11545566 G allele had a significantly elevated CAD risk compared to those of non-carriers [GG vs. AA: adjusted odds ratio (AOR) = 1.62, 95% confidence interval (CI) = 1.13-2.32, P = 0.008; GA/GG vs. AA: AOR = 1.67, 95% CI = 1.22-2.29, P = 0.001]. The genotype frequencies of rs10913733 (35.2% for TT, 46.7% for GT and 18.1% for GG) among CAD patients were significantly different from those (42.1% for TT, 44.3% for GT and 13.6% for GG) among health controls (P = 0.014). After adjustment for age, sex, hypertension, diabetes, smoking, drinking status, BMI, TG, TC, HDL-C, LDL-C and Glucose, carriers of rs10913733 G allele had a significantly elevated CAD risk compared to those of non-carriers (GG vs. TT: AOR = 1.57, 95% CI = 1.08-2.28, P = 0.018; GT/GG vs. TT: AOR = 1.39, 95% CI = 1.07-1.79, P = 0.013). However, There were no significant differences between CAD patients and health controls in the distribution of rs1044925 and rs12121758 genotypes ( P = 0.867 and P = 0.592, respectively). Used multiple comparison, sixteen tests were performed, a significance level was P < 0.003125 (0.05/16) based on Bonferroni correction. We found thatrs11545566 still influenced the risk of Coronary heart disease under the dominantmodel (GA/GG vs. AA: AOR = 1.67, 95% CI = 1.22-2.29, P = 0.001).

**Table 2:** The distribution of genotypes in ACAT-1 gene between CAD patients and controls

Genotype	Model		Case (*n*, %)	Control (*n*, %)	*P*	Crude OR (95% CI)	*P*	Adjusted OR (95% CI)	*P*^a^
rs1044925	Codominant	AA	590(78.7)	450(77.6)	0.867	1	0.867	1	0.686
		AC	149(19.9)	122(21.0)		0.932(0.712-1.219)	0.606	0.929(0.683-1.264)	0.64
		CC	11(1.5)	8(1.4)		1.049(0.418-2.629)	0.919	1.477(0.499-4.370)	0.481
	Dominant	AA	590(78.7)	450(77.6)	0.636	1	0.636	1	0.769
		A/C-C/C	160(21.3)	130(22.4)		0.939(0.722-1.220)		0.956(0.708-1.290)	
	Recessive	A/C-A/A	739(98.5)	572(98.6)	0.894	1	0.894	1	0.464
		CC	11(1.5)	8(1.4)		1.064(0.425-2.663)		1.499(0.508-4.425)	
	Overdominant	A/A-C/C	601(80.1)	458(79.0)	0.6	1	0.6	1	0.611
		AC	149(19.9)	122(21.0)		0.931(0.712-1.217)		0.924(0.680-1.225)	
rs11545566	Codominant	AA	128(17.1)	133(22.9)	0.028	1	0.029	1	0.006
		GA	377(50.3)	272(46.9)		1.440(1.079-1.922)	0.013	1.706(1.222-2.381)	0.002
		GG	245(32.7)	175(30.2)		1.455(1.066-1.985)	0.018	1.618(1.131-2.315)	0.008
	Dominant	AA	128(17.1)	133(22.9)	0.008	1	0.008	1	0.001
		G/A-G/G	622(82.9)	447(77.1)		1.446(1.102-1.897)		1.671(1.220-2.287)	
	Recessive	G/A-A/A	505(67.3)	405(69.8)	0.332	1	0.332	1	0.465
		GG	245(32.7)	175(30.2)		1.123(0.889-1.419)		1.105(0.846-1.442)	
	Overdominant	G/G-A/A	373(49.7)	308(53.1)	0.223	1	0.223	1	0.062
		GA	377(50.3)	272(46.9)		1.144(0.921-1.422)		1.267(0.988-1.625)	
rs12121758	Codominant	CC	227(30.3)	190(32.8)	0.592	1	0.592	1	0.456
		AC	388(51.7)	293(50.5)		1.108(0.867-1.416)	0.411	1.172(0.884-1.554)	0.27
		AA	135(18.0)	97(16.7)		1.165(0.842-1.611)	0.356	1.219(0.843-1.762)	0.293
	Dominant	CC	227(30.3)	190(32.8)	0.331	1	0.331	1	0.217
		A/C-A/A	523(69.7)	390(67.2)		1.122(0.889-1.417)		1.184(0.905-1.548)	
	Recessive	A/C-C/C	615(82.0)	483(83.3)	0.543	1	0.543	1	0.552
		AA	135(18.0)	97(16.7)		1.093(0.820-1.456)		1.103(0.798-1.524)	
	Overdominant	A/A-C/C	362(48.3)	287(49.5)	0.66	1	0.66	1	0.496
		AC	388(51.7)	293(50.5)		1.050(0.845-1.304)		1.090(0.851-1.396)	
rs10913733	Codominant	TT	264(35.2)	244(42.1)	0.014	1	0.014	1	0.03
		GT	350(46.7)	257(44.3)		1.259(0.993-1.595)	0.057	1.330(1.012-1.747)	0.041
		GG	136(18.1)	79(13.6)		1.591(1.147-2.207)	0.005	1.569(1.081-2.278)	0.018
	Dominant	TT	264(35.2)	244(42.1)	0.011	1	0.011	1	0.013
		G/T-G/G	486(64.8)	336(57.9)		1.337(1.070-1.670)		1.388(1.073-1.794)	
	Recessive	G/T-T/T	614(81.9)	501(86.4)	0.027	1	0.027	1	0.093
		GG	136(18.1)	79(13.6)		1.405(1.039-1.899)		1.339(0.953-1.881)	
	Overdominant	T/T-G/G	400(53.3)	323(55.7)	0.392	1	0.392	1	0.241
		GT	350(46.7)	257(44.3)		1.100(0.885-1.367)		1.160(0.905-1.488)	

^a^ Adjusted for age, sex, hypertension, diabetes, smoking, drinking status, BMI, TG, TC, HDL-C, LDL-C, Glucose.

*p* < 0.05 indicates statistical significance.

*p* < 0.003125 indicates statistical significance for multiple comparison.

**Figure 1:**
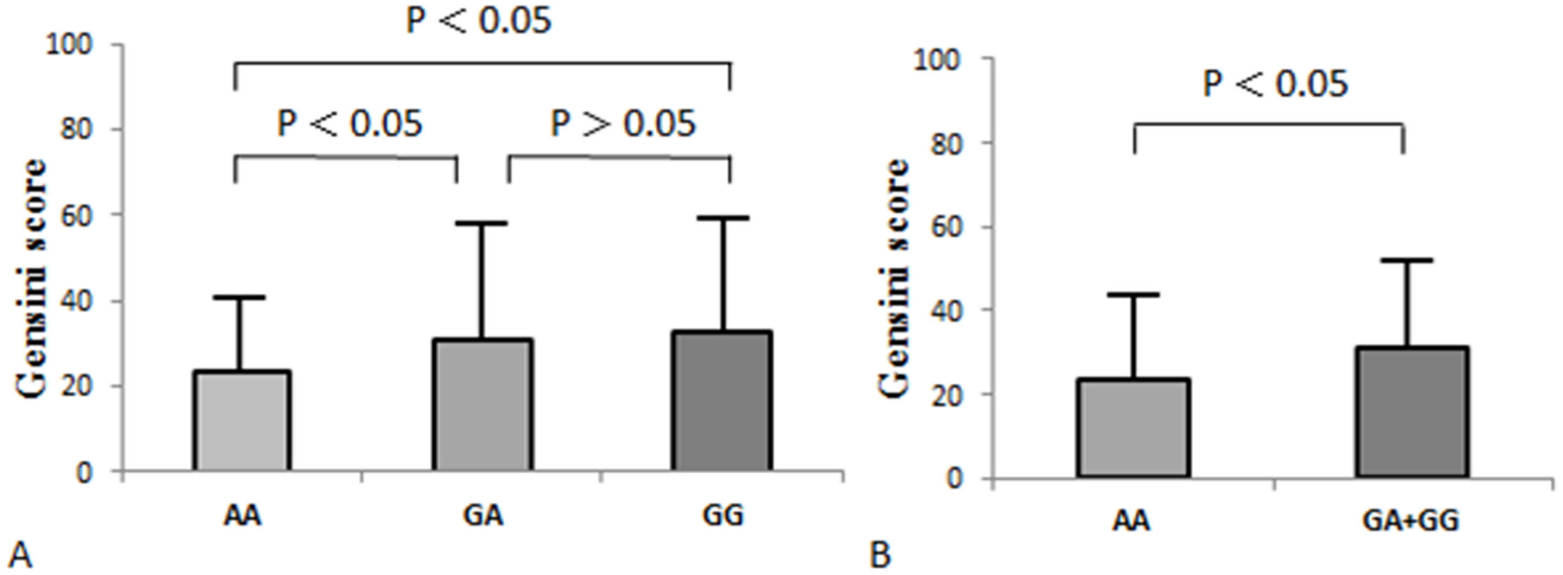
Influence of the rs11545566 polymorphisms on Gensini score in the CAD patients **(A)** Comparison among different genotypes of rs11545566. **(B)** Comparison between patients with GG/GA genotype and AA genotype.

Furthermore, the association between the 4 polymorphisms and the severity of coronary artery lesions was analyzed among CAD patients. For rs11545566, the Gensini score of subjects in the variant GG genotype group and the GG/GA genotype group were greater than the score of subjects in the AA genotype group (32.49 ± 26.60 and 31.26 ± 26.96 vs. 23.45 ± 21.64; P = 0.001 and 0.002, respectively) (Figure 1). Rs11545566 was found to be independently associated with the Gensini scores after adjusting for other confounding factors (P = 0.005). But there existed no significantly association between rs1044925, rs12121758, rs10913733 and Gensini scores (P > 0.05).

### Stratified analysis

We performed stratification analyses in terms of age, gender, smoking status, drinking status, hypertension and diabetes to evaluate how these variables modified the association between the SNPs (rs11545566, rs10913733, rs1044925 and rs12121758) and CAD risk (Table 3). The rs11545566 GA/GG genotypes were shown to significantly increase CAD risk in males (AOR = 1.61, 95% CI = 1.10-2.38), nonsmokers (AOR = 1.91, 95% CI = 1.30-2.81), nondrinkers (AOR = 1.85, 95%CI = 1.29-2.66), subgroup with hypertension (AOR =1.97, 95% = 1.28-3.01) and subgroup without diabetes (AOR = 1.57, 95% CI = 1.10-2.43). The rs12121758 AC/AA genotypes were shown to significantly increase CAD risk in subjects of age>61 (AOR = 1.57, 95% CI = 1.06-2.33). The rs10913733 GT/GG genotypes were shown to significantly increase CAD risk in subjects of age>61 (AOR = 1.89, 95% CI = 1.30-2.75), females (AOR = 1.86, 95% CI = 1.16-2.98), nonsmokers (AOR = 1.50, 95% CI = 1.10-2.04), nondrinkers (AOR = 1.44, 95%CI = 1.07-1.94), subgroup with hypertension (AOR =1.75, 95% = 1.24-2.48) and subgroup without diabetes (AOR = 1.39, 95% CI = 1.04-1.86).

**Table 3:** Stratifed analysis between ACAT-1 gene polymorphism and CAD risk

Variables	rs1044925 (case/control)		Adjusted Ora	*P*^a^	rs11545566 (case/control)		Adjusted Ora	*P*^a^	rs12121758 ( control/case)		Adjusted Ora	*P*^a^	rs10913733 (case/control)		Adjusted Ora	*P*^a^
	AA	A/C-C/C	95% CI		AA	G/A-G/G	95% CI		CC	A/C-A/A			TT	G/T-G/G		
**Median age, yr**																
≤61	302/240	87/67	1.024(0.673-1.558)	0.913	69/62	320/245	1.477(0.939-2.324)	0.092	126/88	263/219	0.885(0.607-1.290)	0.526	144/114	245/193	1.015(0.707-1.456)	0.936
>61	288/210	73/63	0.892(0.575-1.384)	0.611	59/71	302/202	1.911(1.221-2.991)	1.911	101/102	260/171	1.571(1.061-2.326)	0.024	120/130	241/143	1.891(1.300-2.751)	0.001
**Gender**																
**Females**	175/140	36/39	0.813(0.462-1.432)	0.473	35/44	176/135	1.720(0.987-2.998)	0.056	57/61	154/118	1.247(0.756-2.056)	0.388	66/84	145/95	1.857(1.159-2.976)	0.01
**Males**	415/310	124/91	1.006(0.699-1.449)	0.972	93/89	446/312	1.615(1.097-2.377)	0.015	170/129	269/272	1.113(0.804-1.543)	0.518	198/160	341/241	1.232(0.899-1.686)	0.194
**Smoking status**																
**Never**	328/364	65/110	0.910(0.636-1.302)	0.605	63/111	350/363	1.910(1.300-2.806)	0.001	114/160	299/314	1.304(0.942-1.807)	0.11	135/203	278/271	1.497(1.098-2.042)	0.011
**Ever**	262/86	75/20	1.305(0.717-2.374)	0.384	65/22	272/84	1.256(0.698-2.259)	0.447	113/30	224/76	0.841(0.504-1.404)	0.508	129/41	208/65	1.117(0.687-1.815)	0.655
**Drinking status**																
**Never**	429/352	100/109	0.824(0581-1.168)	0.277	93/112	436/349	1.852(1.291-2.656)	0.001	153/159	376/302	1.341(0.982-1.831)	0.065	191/203	338/258	1.442(1.072-1.938)	0.015
**Ever**	161/98	60/21	1.860(0.951-3.637)	0.07	35/21	186/98	1.110(0.554-2.222)	0.769	74/31	147/88	0.681(0.382-1.216)	0.194	73/41	148/78	1.164(0.667-2.030)	0.593
**Hypertension**																
**No**	218/259	52/71	1.036(0.652-1.646)	0.882	52/74	218/256	1.339(0.843-2.127)	0.217	81/101	189/229	1.071(0.540-1.171)	0.246	101/128	169/202	1.037(0.702-1.531)	0.856
**Yes**	372/191	108/59	0.925(0.620-1.380)	0.704	76/59	404/191	1.966(1.284-3.010)	0.002	146/89	334/161	1.298(0.907-1.859)	1.298	163/116	317/134	1.754(1.241-2.479)	0.001
**Diabetes**																
**No**	407/392	107/115	0.911(0.652-1.272)	0.584	84/113	430/394	1.573(1.103-2.243)	0.012	151/164	363/343	1.173(0.869-1.584)	0.297	172/209	342/298	1.392(1.044-1.857)	0.024
**Yes**	183/58	53/15	1.128(0.543-2.345)	0.746	44/20	192/53	1.952(0.979-3.890)	0.57	76/26	160/47	1.074(0.572-2.015)	0.825	92/35	144/38	1.287(0.708-2.340)	0.407

^a^ Adjusted for age, sex, hypertension, diabetes, smoking, drinking status, BMI, TG, TC, HDL-C, LDL-C, Glucose.

*p* < 0.05 indicates statistical significance.

## DISCUSSION

In the present study, we investigated associations between four SNPs in the SOAT-1 gene and CAD risk in a Chinese Han population. Our results indicate that rs11545566 and rs10913733 were strongly correlated with CAD susceptibility.

Cholesterol is an important structural component of cell membranes, and a precursor for bile acids, vitamin D, and steroid hormone [20]. Substantial studies have suggested that high level of blood cholesterol is closely related to an increase risk of CAD, which is becoming the leading cause of death in developed countries [21–22]. Atherosclerosis is a chronic disease characterized by the deposition of excessive cholesterol in the arterial intima [23]. The initial stage of atherosclerosis is promoted by cholesterol ester (CE) accumulation in macrophages [24]. ACAT-1 is an integral membrane protein that converts free cholesterol into the storage form of CE [11]. Previous studies have suggested that deficiency of ACAT-1 gene could increase the synthesis and efflux of free cholesterol in mouse macrophages and enhancement of human ACAT-1 gene expression could promote CE accumulation and macrophage-derived foam cell formation [25–26]. Thus, a great deal of researchers believed that ACAT-1 could control the ratio of cellular free cholesterol and CEs [10–12]. However, the role of ACAT-1 in atherosclerosis is currently inconsistent in different studies. Kusunoki et al found that partial ACAT inhibition by F-1394 could decrease early atherosclerosis development in apoE-deficient mice which is a mouse model of atherosclerosis [27]. Further, Rong JX investigated the effects of F-1394 on pre-established, advanced lesions of apoE-deficient mice and found that partial ACAT inhibition by F-1394 lowered plaque cholesterol content and had other anti-thermogenic effects in advanced lesions in apoE-deficient mice without obvious toxicity [28]. Terasaka N et al also found that ACAT inhibitor pactimibe sulfate (CS-505) could not only reduce but also stabilize atherosclerotic lesions by cholesterol-lowering and direct effects in apoE-deficient mice [29]. All these suggested that partial ACAT inhibition may have therapeutic potential in the clinical treatment of atherosclerosis.

While, several studies in animals have also suggested that ACAT inhibitors could accelerate atherosclerosis. Fazio S et al showed that ACAT1-deficient macrophages unexpectedly develop larger atherosclerotic lesions than control in hypercholesterolemic LDL receptor-deficient (LDLR(-/-)) mice model [30]. Su YR et al performed a study in animal and found that deficiency of macrophage ACAT-1 accelerates atherosclerosis in hypercholesterolemic apoE-/- mice [31]. This may be due to the cytotoxic effects of increased free cholesterol levels, which crystallize in macrophage foam cells. Taken together, it is debatable whether the development of macrophages into cholesterol ester–rich foam cells promotes or inhibits atherosclerotic lesion development; the effects of ACAT-1 on atherosclerosis remain to be further investigated.

The associations between ACAT-1 gene polymorphisms and CAD and serum lipid levels have been reported in several previous studies. Ohta T et al performed a study in 178 unrelated normolipidemic and 441 unrelated hyperlipidemic subjects and found that -77G > A variant in ACAT-1 gene were significantly associated with plasma concentrations of HDL-C and apoA-I, but there existed no significantly association between R526G variant in ACAT-1 gene and plasma concentrations of lipids or apolipoproteins [18]. Dong-Feng Wu et al also performed a research regarding the relationship between rs1044925 polymorphism in ACAT-1 gene and CAD [19]. 626 subjects of Bai Ku Yao and 624 subjects of Chinese Han were randomly selected and genotyped in their study and the levels of TC, LDL-C and ApoB were significant difference between genotypes in Bai Ku Yao but not in Han subjects. The C allele carriers had lower levels of TC, LDL-C and ApoB compared with the C allele noncarriers in females in Bai Ku Yao subjects. Furthermore, they performed another study in 1730 unrelated subjects to identify the association between rs1044925 polymorphism in ACAT-1 gene and the risk of CAD and ischemic stroke [32]. They found that the C allele carriers of ACAT-1 rs1044925 had higher serum HDL-C level and the risk of CAD may increase with the presence of the C allele.

However, we found no significant association between ACAT-1 rs1044925 and the risk of CAD in the present study and this was inconsist with previous studies. The reason for this discrepancy is unclear but may be a result of ethnic differences, environmental factors or our relatively small-scale study. We also found that ACAT-1 rs11545566 and rs10913733 were associated with the increased susceptibility to coronary artery disease. In addition, Rs11545566 was also associated with the severity of CAD assessed by Gensini scores.

Despite the promising findings in this study, some inherent limitations of this case-control study must be noted. First of all, due to the transversal character of the present study, we failed to get a cause-and-effect relationship between risk factors and CAD. Secondly, all the samples were from the Chinese Han population living in Xinjiang and from the same hospital. There exists potential confounding factors which may have caused type I errors (false-positive results) in our study. Thirdly, some positive SNPs identified in previous studies became negative in the present study. The reason for this discrepancy is unclear but possible explanation may be that different populations have different lifestyles, modifier genes, gene-gene and gene-environment interactions. Therefore, further large-scale investigations should be carried out on different ethnic populations.

In summary, our findings provide support for an independent leading contribution of rs11545566 and rs10913733 in ACAT-1 gene to CAD risk in Han Chinese. However, the precise functions of these polymorphisms have yet to be determined. Additional studies need to be undertaken to clarify the underlying molecular mechanism that associates the ACAT-1 polymorphisms with CAD.

## MATERIALS AND METHODS

### Ethical approval of the study protocol

This study was approved by the Ethics Committee of the First Affiliated Hospital of Xinjiang Medical University (Xinjiang, China). It was conducted according to the standards of the Declaration of Helsinki. All of the patients provided written informed consent and explicitly provided permission for DNA analyses, as well as for the collection of relevant clinical data.

### Subjects

All of the participants were Han Chinese. Between 2010 and 2016, we recruited 750 CAD patients and 580 health controls at the First Affiliated Hospital of Xinjiang Medical University. The definition of CAD was defined as presence of at least one significant coronary artery stenosis of > 50% luminal diameter based on the coronary angiography. Exclusion criteria were those with concomitant valvar heart disease, congenital heart disease, and/or nonischemic cardiomyopathy. We randomly selected 580 age and sex matched participants as the control group. All control subjects were selected from volunteers who had visited our hospital between 2010 and 2016 for regular medical check-ups and were found to be healthy. Individuals would be considered eligible disease-free controls if they had angiographically normal coronary arteries and had no history of CAD [33]. Coronary angiography in the control individuals was performed for the evaluation of chest pain. Individuals were excluded from this study if they had: a history of CAD; electrocardiographic signs of CAD; regional wall motion abnormalities; relevant valvar abnormalities in echocardiograms and/or carotid atherogenesis. The response rates of participants were 100%.

### Laboratory examination and definition of cardiovascular risk factors

Serum concentrations of TC, TG, HDL-C, LDL-C and Glucose were measured using standard methods in the Department of Clinical Laboratory of First Affiliated Hospital, Xinjiang Medical University as described previously [34, 35]. Hypertension was defined as self-reported use of antihypertensive medication within the past 2 weeks or an average systolic blood pressure ≥ 140 mm Hg, an average diastolic blood pressure ≥ 90 mm Hg, or both [36]. Briefly, diabetes was defined as fasting plasma glucose ≥ 6.99 mmol/L, the use of insulin or oral hypoglycemic agents, or a self-reported history of diabetes [37]. Smoking was defined as currently smoking cigarettes.

### Coronary angiography

Coronary angiography was performed in all CAD patients. Angiographic evaluations were reviewed by 3 independent interventional cardiologists blinded to the study information. In case of disagreement, the decision was based on the judgment of the fourth, more experienced cardiologist.

Gensini score: angiographic stenosis of a culprit artery in the range of 0% to 25% was scored as 1 point, stenosis in the range of 25% to 50% was scored as 2 points, 50% to 75% was scored as 4 points, 75% to 90% was scored as 8 points, 90% to 99% was scored as 16 points, and total occlusion was scored as 32 points. A multiplier was assigned to each main vascular segment based on the functional significance of the myocardial area supplied by that segment: 5 for the left main coronary artery, 2.5 for the proximal segment of the left anterior descending (LAD) coronary artery and the proximal segment of the circumflex artery, 1.5 for the mid-segment of the LAD, 1.0 for the right coronary artery, the distal segment of the LAD, mid-distal region of the circumflex artery, the posterolateral artery, and the obtuse marginal artery, and 0.5 for other segments [38].

### Genotyping of ACAT-1 gene

Using Haploview 4.2 software and International HapMap Project website phase I &II database (http://www.hapmap.org), we obtained four tag SNPs of ACAT-1: SNP1 (rs1044925), SNP2 (rs11545566), SNP3 (rs12121758) and SNP4 (rs10913733) by using minor allele frequency (MAF) ≥0.05 and linkage disequilibrium patterns with r2≥0.8 as a cutoff. The basic information of SNPs were showed in Table 4. Blood samples were collected from all participants using a standard venipuncture technique and EDTA containing tubes. DNA was extracted from the peripheral blood leukocytes using a whole blood genome extraction kit (Beijing Bioteke Corporation, Beijing, China). The SNP genotyping was performed using an improved multiplex ligation detection reaction (iMLDR) technique (Genesky Biotechnologies Inc., Shanghai, China). The primers for the polymerase chain reaction (PCR) and the probes for the LDR were listed in Supplementary Table 1. Genotyping was performed in a blinded fashion without knowledge of the patients’ clinical data, and a total of 10% of the genotyped samples were duplicated to monitor genotyping quality.

**Table 4:** Basic SNPs in ACAT-1 gene summary of all study participants

SNP ID	Alleles	Chromosome	Position	Band	MAF control	MAF case	OR(95%CI)	*P* value
rs1044925	A/C	1	179354603	1q25.2	0.119	0.114	0.95(0.75-1.21)	0.692
rs11545566	A/G	1	179293868	1q25.2	0.464	0.422	1.19(1.02-1.38)	0.031
rs12121758	A/C	1	179355735	1q25.2	0.42	0.439	0.93(0.79-1.08)	0.33
rs10913733	G/T	1	179347934	1q25.2	0.358	0.415	2.53(2.16-2.97)	<0.001

### Statistical analysis

The data analysis was performed using SPSS version 17.0 for Windows (SPSS Inc., Chicago, IL, USA). The Hardy-Weinberg equilibrium was assessed by Chi-square test. The measurement data are shown as the means±SD, and the differences between the CAD patients and health control were assessed using an independent-sample t-test. Differences in the enumeration data, such as the frequencies of smoking, drinking, hypertension and genotypes between the CAD patients and health control were analyzed using the chi-square test. Additionally, logistic regression analyses with effect ratios (OR and 95% CI) were used to assess the contribution of the major risk factors to CAD. Multivariate analysis was conducted after adjustment for age, gender, smoking, drinking status, hypertension, diabetes, BMI, glucose, TG, TC, HDL-C and LDL-C. Linear regression models were constructed to test the additive effects of the SNPs on the severity of CAD which was assessed by Gensini scores. A 2-tailed P value less than 0.05 was considered to be statistically significant. We also performed Bonferroni correction to assess the effect of genetic polymorphisms on coronary heart disease.

## SUPPLEMENTARY MATERIALS TABLE


