# Supplementary material for: ACAT-1 gene polymorphism is associated with increased susceptibility to coronary artery disease in Chinese Han population: a case-control study
Source: Oncotarget. 2017 Oct 6;8(51):89055–63. doi: 10.18632/oncotarget.21649 (PMC5687668; doi:10.18632/oncotarget.21649)
Supplement: Supplementary file 1 [file oncotarget-08-89055-s001.pdf]

ACAT-1 gene polymorphism is associated with increased susceptibility to coronary artery disease in Chinese Han population: a case-control study

SUPPLEMENTARY MATERIALS

Supplementary Table 1: The primers for the polymerase chain reaction (PCR) and the probes for the LDR

SNPs	Primers or probes	Sequences
rs1044925	Upstream primer	GGAAGGAAAAAATAAGAGATAGCAGAG
	Downstream primer	TGATCACTTTAATATGAGTGGAGGTGA
	RC	TCTCTCGGGTCAATTCGTCCTTTGATCACTTTAATATGAGTGGAGGTGATTAATACTTAAGG
	RA	TGTTCGTGGGCCGGATTAGTTGATCACTTTAATATGAGTGGAGGTGATTAATACTTAGGT
	RP	TACAGACTCACTAGTCTGCAAAATAATTCAAGTTTTTTTTTTTTTTTTTTTTTTTTTTTTTT
rs11545566	Upstream primer	AACCTGGGGACCACCAATAGGA
	Downstream primer	ACTTCGGCCAAGAGGGCAGAG
	FG	TTCCGCGTTCGGACTGATATCGGAGTCGACCTTCCTGCAGG
	FA	TACGGTTATTCGGGCTCCTGTCGGAGTCGACCTTCCTGCAGA
	FP	CTGCTCTGTGACCGCTTCCCTTTTTTTTTTT
rs12121758	Upstream primer	GGACAGATTGCGCTGAGACCTG
	Downstream primer	GTTGCAATCTTGGGCAGGAAAA
	FC	TTCCGCGTTCGGACTGATATCTAATTTTTGTATTTTTAGTAGAGATGGGGTTTCAACC
	FA	TACGGTTATTCGGGCTCCTGTCTAATTTTTGTATTTTTAGTAGAGATGGGGTTTCAGCA
	FP	tgttggtcaggctggtctcaATTTTTTTTTTTTTTTTTTTTTTTT
rs10913733	Upstream primer	TGGCTAATGTCACCAGCCTTTCA
	Downstream primer	TCAGGGAAGTCTCATGGTGTGCT
	RG	TTCCGCGTTCGGACTGATATAATCACCAATATATCCTTGCAGGATACC
	RT	TACGGTTATTCGGGCTCCTGTAATCACCAATATATCCTTGCAGGATGCA
	RP	GCAGAAACCATGTGCTTATGTTAAGGTTTTTTTTTTTTTTTTTTTTT
